# Is there a role for microbiome-based approach in common variable immunodeficiency?

**DOI:** 10.1007/s10238-023-01006-3

**Published:** 2023-02-03

**Authors:** Remo Poto, Gianluca laniro, Amato de Paulis, Giuseppe Spadaro, Gianni Marone, Antonio Gasbarrini, Gilda Varricchi

**Affiliations:** 1 Department of Translational Medical Sciences, University of Naples Federico II, 80131 Naples, Italy; 2 Center for Basic and Clinical Immunology Research (CISI), University of Naples Federico II, 80131 Naples, Italy; 3 World Allergy Organization (WAO), Center of Excellence, 80131 Naples, Italy; 4 Department of Oncology and Molecular Medicine, Istituto Superiore Di Sanità (ISS), Rome, Italy; 5 Digestive Disease Center, Fondazione Policlinico Universitario “A. Gemelli” IRCCS, Rome, Italy; 6 Department of Translational Medicine and Surgery, Catholic University of Rome, Rome, Italy; 7 Institute of Experimental Endocrinology and Oncology (IEOS), National Research Council, 80131 Naples, Italy

**Keywords:** Common variable immunodeficiency, Fecal microbiota transplantation, Gut microbiome, Microbiome-based therapies, Personalized medicine

## Abstract

Common variable immunodeficiency (CVID) is a primary immunodeficiency characterized by low levels of serum immunoglobulins and increased susceptibility to infections, autoimmune disorders and cancer. CVID embraces a plethora of heterogeneous manifestations linked to complex immune dysregulation. While CVID is thought to be due to genetic defects, the exact cause of this immune disorder is unknown in the large majority of cases. Compelling evidences support a linkage between the gut microbiome and the CVID pathogenesis, therefore a potential for microbiome-based treatments to be a therapeutic pathway for this disorder. Here we discuss the potential of treating CVID patients by developing a gut microbiome-based personalized approach, including diet, prebiotics, probiotics, postbiotics and fecal microbiota transplantation. We also highlight the need for a better understanding of microbiota-host interactions in CVID patients to prime the development of improved preventive strategies and specific therapeutic targets.

## Introduction

The human microbiome consists of approximately 10–100 trillion microbial cells harbored by an individual, the majority of which live in the gut [1]. The gut microbiome has evolved with the host species over thousands of years to form a complex and mutually beneficial relationship. The composition of gut microbiota is a dynamic process changing throughout life. Microbiome establishment begins with vertical transmission of maternal microbiome at birth [2]. The colonization of gut microbiome during the early stages of life plays a crucial role in its future composition [3]. Multiple variables can influence gut microbial composition, including mode of delivery, early feeding, antibiotic use, diet and environmental factors [2, 4–6]. The gut microbiome plays a crucial role in maintaining immune homeostasis and modulating the host's innate and adaptive immune response [7–10]. It is also crucial for maintaining gut epithelial barrier homeostasis and orchestrating defense against pathogens [11]. Hence, a eubiotic gut microbiota is essential in maintaining human health and preventing diseases. Gut microbiota dysregulation is suggested to play a key role in the development of several disorders, including inflammatory bowel diseases [12], irritable bowel syndrome [13], metabolic diseases [14], autoimmune disorders [15] and cancer [16, 17]. Gut microbiota profiling and modulation (e.g., diet, prebiotics, probiotics, postbiotics and fecal microbiota transplantation) may thus represent a promising tool to manage these disorders. Prebiotics are non-digestible fibers that selectively stimulates the growth and/or activity of indigenous bacteria [18]. Probiotics are live microorganisms which when administered in adequate amounts confer a health benefit to the host [19]. Postbiotics are preparation of inanimate microorganisms and/or their components that physiological benefits to the host [20] and are produced from inactivated commensal bacteria.

Common variable immunodeficiency (CVID) is the most common symptomatic primary immune deficiency (PID) in adulthood and is characterized by low levels of serum immunoglobulins (IgG and IgA, with or without IgM) and impaired antibody production in response to vaccines and pathogens [21, 22]. CVID encompasses a broad spectrum of heterogeneous manifestations related to complex immune dysregulation. Although the increasing use of next-generation sequencing (NGS) technologies has promoted the discovery of multiple genes associated with specific CVID phenotypes [23, 24], the pathogenesis is complex probably implying the environment, genetic and epigenetic alterations [25]. Emerging evidence highlights that both the intestinal ecosystem and the gut microbiota are profoundly disrupted in patients with CVID [26–29]. Recent evidence indicates that CVID patients with enteropathy have a more marked transcriptional response to gut viruses [30–32]. CVID patients have increased susceptibility to a wide range of infections [33], autoimmune diseases [34–36] and cancers [37–40]. Intravenous (i.v.) or subcutaneous (s.c.) immunoglobulin replacement therapy (IgRT) has been shown to reduce life-threatening infections of CVID patients, radically improving their survival [37]. On the other hand, IgRT has not demonstrated efficacy in preventing and treating complications related to immune dysregulation.

## Gastrointestinal manifestations of CVID

Gastrointestinal (GI) manifestations are common in CVID ranging between 15 and 50% [41–43]. GI complications of CVID can involve any part the gastrointestinal tract, but the most commonly affected sites are the small bowel, the colon, the stomach and the liver [42, 44, 45]. Small bowel villous atrophy can be present mimicking celiac disease, but patients with CVID typically do not respond to a gluten-free diet (GFD) and they do not express the typical HLA genes associated with celiac disease [46]. Atrophic gastritis can lead to pernicious anemia-like syndrome, which increases the risk for gastric adenocarcinoma [44, 47], the leading cause of cancer death in CVID [48, 49]. Enteropathy, a common CVID manifestation, may resemble celiac disease or inflammatory bowel diseases (IBD). CVID and IBD are related because the prevalence of the latter is increased among CVID patients [50, 51]. Chronic small bowel inflammation may occur in up to 12% of CVID patients and is associated with persistent diarrhea, malabsorption, weight loss and steatorrhea [21, 52]. Additional clinical complications include osteoporosis, zinc, vitamin A, D, B12 and D deficiency [52, 53].

More than 60% of patients with CVID have symptoms of small intestinal bacterial overgrowth [54]. Profiling the gut microbiome of patients with CVID has been performed in hopes to identify a possible association between the microbiome [26, 28, 55–57] and development of GI manifestations, autoimmunity, and malignancy in patients with CVID, but these questions remain unanswered. There is clear evidence that the gut microbiota composition is different between CVID patients and healthy individuals, but whether this difference may be contributing to symptom and disease severity remains to be determined [56, 58]. GI manifestations in CVID are often difficult to control because they do not respond to IgRT [47, 51]. CVID-related enteropathy can be associated with villous atrophy, malabsorption and chronic diarrhea [42, 44, 59]. The latter triad often characterizes difficult to treat CVID patients. Microbial dysbiosis may worsen the damage to the gut barrier and lead to the translocation of bacteria or their fragments and metabolites, promoting systemic inflammation and liver injury [58, 60]. In addition, the disruption of beneficial obligate anaerobes in the gut promotes colonization with multidrug-resistant (MDR) pathogens, such as vancomycin-resistant Enterococcus (VRE) and extended-spectrum β-lactamase producing *Enterobacteriaceae* (ESBL-E) [61, 62]. The treatment of autoimmune and lymphoproliferative complications [e.g., autoimmune cytopenias, lymphoid hyperplasia/splenomegaly, granulomatous-lymphocytic interstitial lung disease (GLILD)] often requires immunosuppressive therapies [63], amplifying the disruption of gut microbiota and infectious risk. Moreover, prophylactic, prolonged and/or repeated antimicrobial therapies, determining gut dysbiosis, can contribute to an increased risk for diarrhea [64, 65]. Restoration of the gut microbiota to a eubiotic state plays a critical role in the management of gastrointestinal manifestations in CVID. Hence, gut microbiota modulation may be a potential therapeutic tool in these patients.

Despite the increasing evidence of alteration of gut microbiome in CVID [26, 28, 55–57], therapeutic manipulation has only been explored as a possible target for CVID patients in one study using rifaximin. Mitigating dysbiosis in patients with CVID is a new pathway to be evaluated, potentially impacting clinical outcomes and survival. Different approaches could improve the gut microbiota composition. Dietary fiber modifications, prebiotic and probiotic supplementation have been suggested to be beneficial chronic human diseases, including cardiovascular, metabolic and cancer diseases [66, 67]. However, no studies have explored the effects of these approaches in CVID patients, and the appropriate prebiotic and probiotic combination beneficial for each individual remains poorly understood. The use of antibiotics still needs confirmation, although eubiotic effects have been reported with the use of rifaximin [28, 68]. Finally, modulation of gut microbiota–intestinal barrier interactions is already considered a therapeutic strategy in several diseases [69].

CVID is a heterogeneous condition and clinical manifestations may vary from increased susceptibility to infections to a variety of inflammatory complications. In addition to GI manifestations, several autoimmune disorders [70], interstitial lung disease [71], polyclonal lymphoproliferations [72], and increased risk of malignancy [44, 73] can occur in CVID patients. The hypothetical relationships between these heterogeneous inflammatory manifestations and gut microbioma are largely unknown.

This review aims to provide a critical overview of possible interventions (e.g., diet, prebiotics, probiotics, postbiotics and fecal microbiota transplantation) that should be investigated in CVID patients with gastrointestinal manifestations for the purpose to restore and/or promote a healthy gut microbiome in these patients.

## Dietary and supplementary interventions

### Diet, prebiotics and short-chain fatty acids

Host diet plays a crucial role in modulating the gut microbiota [74–78]. The plasticity of the human microbiome, its integration with the immune system and its responsiveness to diet make it an extremely attractive therapeutic target.

Dietary fibers (DFs) fermented by gut microbiota have a prebiotic action, which selectively promote the growth and/or activity of the intestinal microbiota, especially *Bifidobacteria* and *Lactobacilli* [79, 80], improving human health in chronic diseases and infections [66]. Not all DFs can be digested by gut microbes, and the term “microbiota accessible carbohydrates” (MACs) refers to complex carbohydrates that cannot be digested by the host but are metabolically available to intestinal microbes. Among these dietary compounds are inulin-type fructans (e.g., inulin, oligofructose and fructooligosaccharides), galactans, galactooligosaccharides (GOS), and other heteropolysaccharides [81]. DFs are fermented by intestinal microbiota to release short-chain fatty acids (SCFAs). SCFAs exert pleiotropic biological functions, including an anti-inflammatory response [82], modulation of intestinal epithelial barrier [83] and maintenance of mucosal immune cell activity [84, 85]. In particular, SCAFs increase Foxp3^+^ regulatory cells (Tregs) during mucosal infection in *Candida albicans*-infected mice [86], upregulates IL-10 production and interfere with the production of pro-inflammatory molecules of IL-12, TNF-α, IL-1β and NO by inhibiting NF-κB activity [87–89].

Diets rich in MACs (e.g., whole grains, vegetables, legumes without processed foods) and exposure to fermented foods (a known source of lactic acid bacteria) may be beneficial for preserving gut microbial communities beneficial to human health [90]. DFs also promote the secretion of mucus from the intestinal epithelial barrier and enhance the expression of tight junction proteins. A known consequence of MAC restriction is the use of intestinal mucus as the primary source of energy by intestinal microbiota resulting in disrupted gut barrier integrity and decreased SCFA production. As a result of inadequate MACs, the mucus layer in the colon may be degraded by mucin-degrading bacteria, thus allowing greater epithelial access and the occurrence of lethal colitis by mucosal pathogens [91].

A recent study showed that a fermented-food diet might enhance microbiome diversity and reduce various inflammation markers (i.e., IL-6, IL-10 and IL-12b) in healthy adults [92]. The latter study showed that a high-fiber diet could modulate immune responses and gut microbiome functions.

In contrast, the Western diet (rich in animal protein and fat, poor in fiber) reduces bacterial diversity and richness, with a significant decline in numbers of beneficial *Bifidobacterium, Lactobacillus* and *Eubacterium* species and an increase in *Bacteroides* and *Enterobacteria*, as compared to a plant-based diet [90].

Diet is a major factor that can shape gut barrier structure and function [93]. Fermented food (i.e., yogurt) confers a benefit on gut epithelial barrier function, as suggested by decreased plasma soluble CD14 (sCD14) concentrations, a surrogate marker of gut microbial translocation [94]. In particular, plasma sCD14 is released by macrophages and hepatocytes as part of the innate immune response to lipopolysaccharide (LPS), a component of gram-negative bacteria, and it has been used as a marker of gut hyperpermeability.

Several studies have demonstrated that the Mediterranean diet, characterized by a higher intake of vegetables over animal proteins, is the most effective diet in maintaining gut microbial diversity [95, 96]. Strict adherence to the Mediterranean diet has been associated with enhanced levels of SCFAs and *Lactobacillus, Bifidobacterium, Eubacteria, Bacteroides* and *Prevotella,* as well as decreases in *Clostridium*. Furthermore, the beneficial impact of diet on the intestinal microbiota could be explained by its ability to improve inflammation and lipid profile.

Reduced serum levels of vitamin A and D are frequently found in CVID patients [97, 98]. The latter vitamins regulate tight junction molecule expression in the gut barrier [99] and the mucosal immune system, modulating gut microbial species [100, 101]. In addition, vitamin D supplementation promotes gut microbiota richness and reduces the *Firmicutes/Bacteroidetes* ratio [102].

Regarding dietary management of CVID enteropathy, gluten-free diet (GFD) and gluten sensitivity are still debated in CVID patients. CD19 deficiency is a risk factor for monogenic CVID in humans [103]. A mouse model of CVID (CD19^−/−^ mice) shows altered gut microbiota composition and intestinal malabsorption [104]. An elegant study demonstrated that metronidazole and GFD histologically reduced malabsorption of the intestinal mucosa in CD19^−/−^ mice [104]. According to the authors, malabsorption in CD19^−/−^ mice was both microbiota-dependent and gluten-sensitive. Gluten antigens modified by microbial transglutaminase may enhance their immunogenicity and trigger an inflammatory [104]. GFD is often followed by individuals to alleviate gastrointestinal symptoms. In short-term studies, GFD has been shown to influence the composition and function of the intestinal microbiota in healthy subjects [105, 106]. In patients with celiac disease, the GFD over two years may also alter the gut microbiota profile, decreasing *Bifidobacteria* and *Lactobacilli* and increasing potential *Enterobacteriaceae* pathobionts. Jorgensen and colleagues have debated the clinical relevance of CD19^−/−^ mice as a CVID model [107]. This mouse model does not represent the polygenic etiology of the majority of CVID patients. Therefore, the results derived from animal models should be critically evaluated when investigating the relationship between microbiota and immunodeficiency.

Patients with CVID could be at increased risk of lactose intolerance (LI) because of “sprue-like” enteropathy. Although there is no evidence, it is possible to speculate that CVID patients with enteropathy do not express lactase at the brush border of the small intestine. Hence, secondary LI may contribute to chronic diarrhea in CVID patients. Undigested lactose in the small intestine causes osmotic water trapping, and the colonic osmotic load is increased approximately eightfold by fermentation of lactose to SCFAs. Future studies are needed to evaluate the prevalence of LI in CVID and whether the effects of a lactose-free diet or the use of exogenous digestive enzymes [108] may have a positive impact on gastrointestinal symptoms. In this context, the gut microbiota could be influenced by biotic interventions, which may improve the symptoms and clinical signs of LI [109]. The use of probiotics significantly reduced abdominal pain, bloating and/or flatulence, vomiting and diarrhea in individuals with LI. The latter effect is associated with reduced exhaled H2 [109]. Several mechanisms may explain the above-mentioned effects. First, probiotics can produce lactase in the gastrointestinal tract [110], promoting colonic fermentation and the overall hydrolytic capacity [111]. Second, probiotics inhibit the growth of heterofermentative bacteria (which produce gas), resulting in improved colonic compensation [112] by secreting antimicrobial peptides [113], adhering competitively to the mucosa, and modulating intestinal barrier permeability [114, 115]. Several probiotics (including *L. rhamnosus, L. acidophilus, L. bulgaricus*, *L. reuteri, S. thermophilus* and *B. longum*) have been found to be effective in attenuating clinical signs of LI in populations with altered lactose absorption [109].

## Probiotics

Probiotics are microbial strains that provide health benefits to the host when administered in adequate amounts [19, 116]. Probiotics have a role in maintaining the immune system homeostasis in the gastrointestinal tract as result of direct interactions with several immune cells [117, 118]. Probiotics can be found in fermented foods, either naturally or artificially added, and can colonize the human gastrointestinal tract. Microbiome richness is essential in health maintenance, and broad-spectrum probiotics are helpful in the prevention and therapy of various diseases [119, 120]. The main probiotic products currently on the market are developed with *Lactobacilli*, *Bifidobacteria* and other lactic acid bacteria, such as *Streptococci* and *Lactococci*. Promising probiotic strains include the bacterial genera *Escherichia, Bacillus*, *Propionibacterium* and some other yeast genera, mainly *Saccharomyces*. The first available probiotics contained only one species of microorganism, whereas subsequent products had a larger variety and number of microorganisms. Probiotic efficacy depends on species, dose and disease [121], and the duration of treatment varies according to the clinical indication.

The effective delivery of living bacteria into lymphoid follicles increases mucosal immune responses, as demonstrated by significantly increased levels of sIgA, CD11c^+^ dendritic cells (DCs), CD4^+^ T cells, and IgA^+^ B cells in the intestinal tract in a mouse model [122]. Probiotics inhibit the growth of pathogenic bacteria, competing for nutrients that would otherwise be utilized by pathogens. Probiotics such as *Lactobacillus rhamnosus* strain GG and *L. plantarum* impede the adhesion of enteropathogenic *Escherichia coli* (*E. coli*) to the gastrointestinal tract [123]. There is evidence that *L. acidophilus* or *L. casei* raised lactic acid bacteria with a concomitant reduction in anaerobes and fecal coliforms [124, 125]]. Moreover, Li et al*.* [126] showed that probiotics shift the composition of gut microbiota toward specific beneficial bacteria (i.e., *Oscillibacter* and *Prevotella*). In an experimental model of hepatocellular carcinoma, the latter bacteria produced anti-inflammatory compounds, which subsequently reduced Th17 polarization and promoted the differentiation of anti-inflammatory Treg/type 1 regulatory T (Tr1) cells in the gut [126].

Probiotics have been shown to promote gut barrier integrity by increasing the number of goblet cells that strengthen the mucus layer [127]. Various *Lactobacillus* species promote mucin expression in human intestinal cell lines [128, 129]. VSL#3, a probiotic mixture of *Lactobacillus* and *Bifidobacterium* species, promotes the expression of MUC2, MUC3 and MUC5AC in HT29 cells [130] and increases tight junction protein expression in vitro and in vivo [131]. Furthermore, *L. acidophilus* A4 cell extract intensifies the expression of MUC2 in HT29 cells, and this effect is unrelated to probiotic adhesion to the cell monolayer [132].

Probiotics promote gut barrier integrity by increasing gene expression in tight junction signaling. *L. acidophilus* and *S. thermophilus* inhibited the attachment of enteroinvasive *E. coli* in HT29 and Caco-2 cells by maintaining (ZO-1, actin) or enhancing (occluding, actinin) cytoskeletal and tight junctional protein phosphorylation [133]. *Lactobacillus rhamnosus* GG (LGG) exerts an important role in epithelial cell survival by activating Akt and inhibiting p38 in response to pro-apoptotic signaling pathways [134].

Probiotics modulate mucosal immune responses by the induction of different cytokines (e.g., IFN-γ and TNF-α), which stimulate an adaptive immune response. This effect is related to the probiotic strain itself [135–137]. Probiotics enhance the production of secretory IgA in vitro [138] and in vivo [139], which is one of the properties by which probiotics can support the immune system [19, 140]. Several studies have demonstrated that specific probiotic strains can enhance the humoral immune response to infections. In children with rotavirus-induced diarrhea, administration of *Lactobacillus* GG showed a marked increase in IgA, IgM and IgG levels [141]. In addition, probiotics influence the immune response and activity of natural killer (NK) cells, which fight virus-infected and cancer cells [142]. Administration of *B. bifidum* and *L. acidophilus* La1 increased the IgA response following immunization for *Salmonella typhi*, whereas *Lactobacillus* GG promoted the immune response to the oral rotavirus vaccine. The latter observations suggest that probiotics may improve vaccine efficacy by acting as potential adjuvants [143].

Another relevant property of probiotics is the inhibition of the growth of pathogenic bacteria by synthesizing low molecular weight compounds such as organic acids (i.e., acetic and lactic acids) and large molecular weight antimicrobial compounds called bacteriocins [144]. These compounds display inhibitory effects on gram-negative bacteria, including *H. pylori* [145]. Bacteriocins produced by probiotics are bifidocin B from *B. bifidum* NCFB, lactacin B from *L. acidophilus*, nisin from *Lactococcus lactis*, and plantaricin from *L. plantarum* [146]. Synbiotic therapy with *B. longum* and a prebiotic (Synergy 1) determine the release of defensins from intestinal epithelial cells in patients with ulcerative colitis [147]. Taken together, all this evidence suggests that probiotics improve gut epithelial barrier tightness and integrity by a variety of mechanisms and that mucosal restoration may positively impact the outcomes of disease. On the other hand, specific studies in cohorts of CVID patients are needed to achieve any evidence.

The effects of probiotics on gut microbiota have been extensively studied; however, there is little evidence on the effects of probiotics on the upper respiratory system. Systematic reviews and meta-analyses found a favorable outcome of using probiotics in reducing the episodes of new respiratory infections in children [148, 149]. Below, we report the evidence regarding the efficacy of probiotics, synbiotics and synthetic microbes in the gastrointestinal manifestations of CVID.

### Acute infectious diarrhea

Probiotics are effective in treating acute infectious diarrhea caused by bacteria, but there are inconsistent results for diarrhea caused by viral pathogens [150, 151]. A Cochrane review of 63 RCTs including 8,014 subjects with acute infectious diarrhea found that probiotics significantly decreased the mean duration of diarrhea (25 fewer hours; 95% confidence interval [CI], 16 to 34 fewer hours), reduced the risk of diarrhea lasting more than four days by 59%, and led to approximately one fewer stool on the second day (mean difference = 0.80; 95% CI, 0.45 to 1.14) [152]. In the case of acute infectious diarrhea, probiotics might be helpful if administered at the onset of symptoms and continued for at least one to two weeks after the symptoms have resolved. However, there are no studies evaluating the effects of probiotics in the prevention and treatment of acute infectious diarrhea in CVID patients.

### Antibiotic-associated diarrhea

Despite adequate i.v. or s.c. IgRT, antibiotics are frequently prescribed to control acute infections or prevent infections in most patients with CVID [153]. Antibiotics induce profound alterations of the gut microbiota with reduced gut SCFA concentrations, increase of luminal carbohydrates and colonic bile acids, impaired water absorption and development of bacterial resistance [154, 155]. Antibiotic-associated diarrhea (AAD) is a relevant morbidity associated with antibiotic use. *C. difficile* is predicted to account for approximately 20% of all AAD cases [156]. Minor opportunistic pathogens, such as *Clostridium perfringens*, *Klebsiella pneumonia*, *Klebsiella oxytoca*, *Staphylococcus aureus* and Candida species, have also been related to AAD [157].

Probiotics could counteract the effects of antibiotics in the gastrointestinal tract by directly preventing the growth of pathogens or by inducing relevant alterations in the gut microbiota composition through the synthesis of SCFAs, production of bacteriocins or reducing luminal pH and O_2_ levels. Probiotics might also modulate the composition of bile acids and interact directly with the gut barrier and the immune system to cause an increase in mucosal response and modulation of water and solute transport [158]. Several bacterial species have been studied in clinical trials for relieving AAD, including members of the *Lactobacillus, Lactococcus, Bifidobacterium, Bacillus, Clostridium, Leuconostoc* and *Streptococcus* genera. Among the yeasts, *Saccharomyces boulardii* has also been studied. *Lactobacillus rhamnosus* strain GG and *S. boulardii* strain CNCM I-745 have been most frequently examined [159, 160]. However, which strains are most effective and their appropriate timing and duration of use are still unknown. Although many studies report that probiotics are generally safe, they should be administered in high-risk groups only after careful evaluation of the risk–benefit ratio [161].

### Helicobacter pylori infection

*Helicobacter pylori* (*H. pylori)* infection affects nearly 50% of the worldwide population and can bring digestive and extra digestive consequences [162]. There are few certainties on the effectiveness of probiotics as a complement to antibiotic therapy to improve *H. pylori* eradication rates. A meta-analysis of nine RCTs with 1,163 patients reported that using *Lactobacillus*-containing probiotics combined with antibiotics increased the *H. pylori* eradication rate compared with the placebo [163]. On the other hand, a recent meta-analysis of 21 RCTs involving 3,452 subjects showed that the combined approach of probiotics with antibiotics did not improve *H. pylori* eradication (odds ratio = 1.44; 95% CI, 0.87 to 2.39) compared to the placebo [164].

## Next-generation probiotics

Additionally to traditional probiotics, live microorganisms with defined clinical benefits claims (also called next-generation probiotics or live biotherapeutics products) are explored as therapeutic agents [165]. Next-generation candidate probiotics comprise *Akkermansia, Roseburia, Faecalibacterium* and *Propionibacterium* species [165–167]. The bacterium *Akkermansia muciniphila* has been labeled as a protective factor against the development of colitis and metabolic diseases [168]. This gram-negative, anaerobic, non-spore-forming, non-motile bacterium*,* belonging to the phylum Verrucomicrobia, is recognized as the first next-generation probiotic. Administration of *A. muciniphila* can help protect against certain metabolic diseases [169–172] and inflammatory disorders [173, 174], influence gut permeability [175], and promote the response to cancer immunotherapy [176] in mouse models. The mechanisms by which *A. muciniphila* potentially protects against various diseases in humans remain unclear. It has been shown that this symbiotic intestinal bacterium is an emerging "gatekeeper of the gut," regulating inflammation and gut epithelium integrity [168, 177, 178]. Probiotics containing *A. muciniphila* are currently under development. However, dietary interventions including polyphenols (i.e., green tea, concord grape, cranberry) [179–181], the antidiabetic drug metformin [182], selective antibiotics (i.e., vancomycin) [183] and FMT [184] are strategies that indirectly increase the abundance of *A. muciniphila*. Gut microbiota enriched in *A. muciniphila* reduces bacterial translocation and inflammation, acting as a shield for gut permeability [185]. Indeed, three in vitro studies reported that *A. muciniphila* improved the integrity of the enterocyte monolayer and increased the expression of cell–cell adhesion and tight junction molecules [177, 186]. In addition, long-term *A. muciniphila* administration improved the thickness of the colonic mucus layer about threefold in an accelerated aging mouse model [178]. *A. muciniphila* could induce butyrate-producing bacterial growth and butyrate production [187], immunoglobulin G1 (IgG1) antibodies, antigen-specific T-cell responses and intestinal adaptive immune responses [188]. Hence, we would like to suggest that *A. muciniphila* might reduce inflammation by preserving intestinal epithelium integrity, supporting butyrate-producing bacteria and subsequently preventing microbial translocation in CVID.

*F. prausnitzii* is another commensal that exerts specific anti-inflammatory properties on the gut [189]. The yeast *S. boulardii* has also been extensively studied in the context of inflammation and intestinal barrier dysfunction. In addition, the favorable effects of this yeast are supported by antimicrobial and antitoxin properties and trophic effects on the intestinal barrier [190].

## Postbiotics, small molecule inhibition, and engineered microbes

Downstream signaling pathways and modulation of the effects of microbial-derived metabolites represent a promising source of new potential therapeutic targets [191, 192]. The latter purposes may be achieved through several methods, including supplementation with bioactive compounds (i.e., postbiotics), small molecule inhibition of microbial enzymes or engineered microbes to perform specific functions. One of the experimental outcomes supporting these action modes is the exogenous administration of SCFAs, which improves inflammatory conditions in colitis mouse models [193].

Conversion of tryptophan to tryptamine and indole metabolites by intestinal bacteria plays an important role in maintaining intestinal barrier function [194]. Indole acetic acid produced by *Lactobacilli* during infections causes the release of IL-22, a cytokine that enhances mucosal immune response in mice and restores gut barrier integrity [195–197]. IL-22 has been shown to have metabolic properties, enhancing insulin sensitivity and reducing endotoxemia [197].

Bacterial lysates (BLs), belonging to the family of postbiotics, are obtained by the chemical/mechanical degradation of bacteria. The rationale for their clinical use in the prevention of microbial infection relates to the concept of the “gut-lung axis,” which represents the interplay between gut-associated lymphoid tissue (GALT) and the respiratory immune system [198]. Lyophilized BLs can reach the Peyer’s patches of the small intestine with stimulation of DCs and activation of B and T lymphocytes [199] which migrate within the mucous membrane of the respiratory tract. As a result, the innate immune system is stimulated and IgA are secreted [200].

Intestinal bacteria may also synthesize vitamin B12 and other B complex vitamins, which are frequently deficient in CVID [201]. A yogurt matrix enriched with *L. acidophilus* has been associated with increased vitamin B12 synthesis and reduced anemia prevalence [202].

Another link between a microbial-derived metabolite and human disease is the role of the proatherogenic metabolite trimethylamine-N-oxide (TMAO) in atherosclerotic disease. A recent study demonstrated that patients with CVID had elevated plasma levels of the metabolites TMAO and trimethylamine (TMA) than healthy individuals [29]. TMAO plasma levels correlated with increased LPS and inflammatory markers (i.e., IL-12 and TNF-α) and with gut abundance of *Gammaproteobacteria* [29]. Preclinical studies have reported that inhibition of a specific microbial enzyme responsible for TMA production from L-carnitine (the first step in TMAO synthesis) reduces atherosclerotic plaque development in a mouse model, providing a proof-of-concept for targeting microbial metabolism [203, 204]. Finally, gut microbial metabolites may exert pleiotropic properties in the human host. SCFAs (acetate, propionate and butyrate) can also act as immunomodulatory and anti-inflammatory metabolites [205, 206]. Other SCFAs derived from amino acid catabolism, including valerate, formate and branched-chain fatty acids, play a minor role in gut homeostasis [207]. SCFAs may have differential effects on T-cell-mediated immune responses, promoting the expansion of Treg cells [208]. In particular, butyrate has been found to promote colonic Treg differentiation from *naïve* CD4^+^ T cells upregulating Foxp3 transcription via histone acetylation [209, 210] or by stimulating GPR109A and GPR43 (GPCRs) signaling [211], as well as activating NLRP3, which is crucial for gut environmental stability and epithelial repair [212]. Exploratory studies in well-characterized CVID cohorts are needed to identify microbial metabolites of interest. Despite the lack of evidence, metabolite-based therapeutics offer significant therapeutic promise. In fact, postbiotics may be safer alternatives for immunocompromised subjects such as CVID patients and could avoid the potential disadvantages of probiotics [213].

Engineered microbes [214] might also have a potential role in CVID disease management. In two murine models of gut barrier impairment, oral supplementation with camouflaged probiotics (*E. coli* Nissle 1917) within a yeast membrane β-glucan enriched, significantly prevents the breakdown of gut barrier and shows reduced bacterial translocation and systemic inflammation [122]. As a consequence of the increase in secretory IgA, there was also an increase in other mediators, such as CD11c^+^ DCs, CD3^+^ T cells, CD4^+^ T cells, CD8^+^ T cells, and IgA^+^ B cells [122].

## Antibiotics

Despite appropriate i.v. or s.c. IgRT, the most common clinical manifestations of CVID are recurrent respiratory and gastrointestinal infections [36, 215]. Therefore, a large percentage of CVID patients are treated with antibiotics to control acute infections or as prophylaxis to limit the frequency of infections [153]. Antibiotics also have adverse side effects, including the development of dysbiosis and bacterial resistance [154]. Some antibiotics may also have positive impacts on the gut microbiota. For instance, rifaximin has been shown to have a eubiotic effect by enhancing the abundance of beneficial bacterial species in patients with various gastrointestinal and liver disorders [68]. Preliminary studies indicated that rifaximin lowered plasma endotoxin levels in cirrhotic patients [216, 217]. In contrast, rifaximin had no effect on circulating biomarkers of systemic inflammation (sCD14, sCD25 or LPS), but lowered microbial alpha diversity in CVID patients [28]. In addition, none of the ten major bacteria implicated in differentiating CVID patients from healthy controls, as measured by the CVID-specific dysbiosis index [55], were affected by rifaximin. The CVID-specific dysbiosis index was correlated with circulating markers of systemic inflammation and intestinal permeability. It is possible to speculate that the absence of CVID specific dysbiosis index variation could explain the lack of an anti-inflammatory effect of rifaximin.

## Fecal microbiota transplantation

Fecal microbiota transplantation (FMT) is an emerging therapy that has become established for the treatment of refractory or recurrent *Clostridioides difficile* infection (rCDI) [218, 219]. The use of FMT for additional disease conditions is currently evaluated, and compelling evidence suggests that FMT may be useful for treating a variety of disorders related to gut dysbiosis [220–225].

FMT is the transfer of an entire microbiome from a healthy donor into the intestinal tract of a recipient. The procedure can be performed via enema [226, 227], oral capsule with fecal extracts [228–230], or colonoscopy-guided insertion [230, 231]. However, the route of delivery may bring along several methodological issues [232]. For instance*, Bacteroidetes* can be damaged by gastric juice as opposed to certain *Firmicutes*, which require passage through the upper gastrointestinal tract to become active [233]. Moreover, a lower gastrointestinal route of administration has shown better results than the upper route in CDI [234]. Finally, the number of fecal infusions may be crucial: A single procedure may be adequate for CDI, but not for chronic diseases that require multiple administrations.

FMT’s therapeutic benefits are due to an increased diversity of bacteria, viruses, fungi and archaea that can engraft into the recipient host and improve microbial diversity. FMT may positively influence the treatment of chronic diarrhea and recurrent infections in patients with CVID. This novel therapeutic approach may be a new technique to re-establish the perturbed gut microbiota of CVID patients and subsequently increase microbiota-derived metabolites such as SCFAs. FMT, through the normalization of the gut microbiome, leads to an augmentation of immune defenses against pathogens (i.e., *C. difficile*) [235, 236]. These benefits comprise the secretion of mucin and antimicrobial peptides, as well as the restoration of the disrupted mucosal barrier or the production of secondary bile acids that inhibit the germination of *C. difficile* spores. To date, the experience of FMT in immunocompromised patients is limited to acquired immunodeficiencies (e.g., HIV infection, immunosuppressive and antineoplastic agents, solid organ transplant recipient), in which FMT is an effective treatment for rCDI and has a comparable incidence of serious adverse events as in other immunocompromised patients [237–239]. However, due to the heterogeneity of immunocompromised patients enrolled in the studies, it is not possible to draw conclusions concerning the efficacy and safety of FMT in specific immunosuppressive states [238].

Although increasing evidence concerning the use of FMT in acquired immunodeficiencies is growing [238–241], there is no evidence in CVID, probably due to the perceived risk of bacterial translocation and sepsis, and the role of FMT in improving clinical outcomes in CVID patients remains unexplored. Recently, safety alerts have been issued by regulatory bodies concerning the risk of pathogens being transmitted via FMT [242]. Detailed recommendations have been established to ensure the safety of FMT during the COVID-19 pandemic [243]. However, we foresee a potential role for this promising therapeutic approach in restoring CVID-associated dysbiosis, as well as a future tool for intestinal decolonization of MDR bacteria.

### Practical considerations for introducing FMT in CVID

Several technical and practical challenges exist, including the selection of clinical donors, preparation of standardized bacterial solutions and capsules, route and type of administration, matching of donors and recipients, prevention and treatment of complications. First, the success rate and efficacy of FMT depend on the gut microbial diversity of the donor and have led to the concept of a “super donor” [244]. Numerous studies have identified desirable qualities of a super donor [245–248]. The FMT response depends on both the donor and recipient's immune system and genetic profile; hence, immune screening becomes crucial before FMT. In the context of CVID enteropathy, the engraftment and efficacy of FMT could be influenced by the absence of intestinal mucosal IgA. Tables 1 and 2 represent a proposed interview and a possible screening to select donors for FMT.

The selection of CVID patients for an FMT trial may be particularly challenging, as patients are frequently treated with antibiotics as prophylaxis to reduce infection frequency or control acute infections, potentially nullifying the efficacy of this therapeutic approach. In addition to the challenges with patient selection, in-depth donor screening is similarly warranted to reduce the transmission of microorganisms that may cause adverse infectious events in an already vulnerable host. The potential for COVID-19 transmission through FMT treatment remains unclear, even though no cases have been reported. Moreover, further studies regarding the impact of COVID-19 on donors’ and recipients’ gut microbiomes are urgently needed.

As our knowledge of gut microbiota involvement in CVID constantly evolves, the potential of therapeutic approaches modulating the gut microbiota to improve GI symptoms in CVID patients is expanding. In the foreseeable future, identifying the exact gut microbiota profile in each CVID patient will be an available and intriguing tool to personalize treatments, including customized prebiotics and probiotics, FMT treatments and/or use of narrow-spectrum antibiotics. Hence, targeted treatments for gut microbiota manipulation may be more effective than conventional probiotics or broad-spectrum antibiotics. In addition, early recognition of gut microbiota changes during patient follow-up may help predict CVID patients at high risk of adverse clinical outcomes. Currently, there are no established guidelines concerning the indications and the optimal FMT procedure for patients with CVID in clinical practice.

More stringent criteria for donor screening should be considered for FMT in CVID patients, and fecal specimen preparation with additional precautions is advisable for a better safety profile. Novel routes of administration for FMT, including fecal capsules, lyophilized stools or fecal filtrate transfer (FFT), could minimize current administration challenges with potentially fewer side effects. On the other hand, their efficacy has not yet been demonstrated [249, 250]. The oral FMT administration route using fecal capsules with frozen or freeze-dried material has a number of advantages, including significantly lower volume and easier administration compared to conventional FMT. This result is a reduction in storage space requirements and an increase in patient compliance [249, 251]. FFT (containing bacterial debris, proteins, metabolic products, antimicrobial compounds and nucleotides) results in a bacteria-free solution. Hence, the transfer of “sterile” fecal filtrate might represent an alternative to conventional FMT, particularly for immunocompromised patients, to decrease the risk of pathogen transmission [252].

FMT for CVID patients should ideally be discussed within a multidisciplinary team (MDT) in a personalized approach. An FMT MDT should include clinical immunologists, gastroenterologists and microbiologists, along with involved nursing and allied health professionals.

## Conclusions

**Fig. 1:**
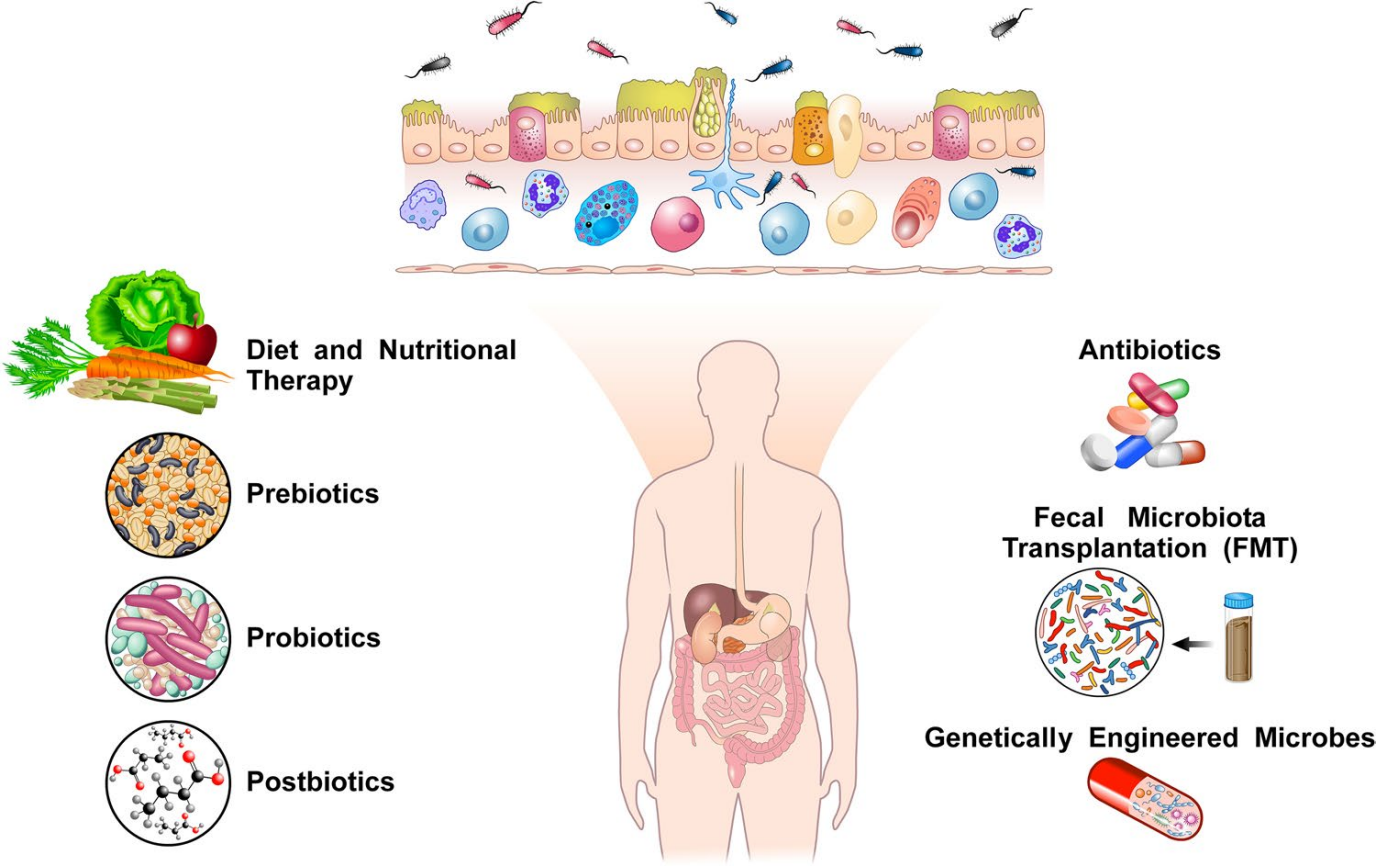
Potential therapeutic approaches to modulate the gut microbiome in CVID patients. Repeated or chronic infections can induce impairment of mucosal immunity with intestinal epithelial damage [253]. Recent evidences suggest that chronic inflammation may impact the gut microbiota [254]. Gut microbial dysbiosis can lead to translocation of bacteria or their fragments and metabolites, promoting activation of innate and adaptive immune cells [58]. It is unknown whether it is possible to improve the clinical outcome of CVID patients with enteropathy through specific therapeutic interventions modulating the gut microbiota. Dietary and nutritional interventions are environmental factors that can modify the gut microbiota composition [74–76]. In particular, high-fiber diets increase beneficial microbes that produce short-chain fatty acids (SCFAs). Other approaches to reverse gut dysbiosis and restore homeostasis include the administration of prebiotics, probiotics or synbiotics [18, 255]. Prebiotics (e.g., oligosaccharides) are nonviable substances that facilitate the growth or activity of specific bacteria. Probiotics comprise individuals or combinations of bacteria. Synbiotics are mixtures of prebiotics and probiotics. Postbiotics (e.g., SCFA) are microbe-derived soluble products and metabolites. Fecal microbiota transplant (FMT), introducing a new bacterial community to the recipient, aiming to reverse the established dysbiosis, might be an appealing therapeutic tool in selected CVID patients. Finally, genetically engineered modified bacteria, that express therapeutic factors into the gut microbiota, might be used for managing pathologies linked to gut microbiome in CVID patients (Tables 1, 2)

There is compelling evidence that the gut microbiome plays a pivotal role in the pathophysiology of CVID. There is the possibility that modulation of the gut microbiota via prebiotics, probiotics and FMT to improve gastrointestinal symptoms could represent a novel treatment strategy in selected patients with CVID. The experimental and clinical development of microbiota modulators in CVID requires a multidisciplinary approach involving translational research teams. Studies in experimental models of CVID appear necessary to better understand the immunological and biochemical effects of modulators of the gut microbiota. Figure 1 summarizes the potential approaches that may modulate the gut microbiome in CVID patients. Future randomized clinical trials should systematically evaluate the potential benefits and risks associated with the use of microbiome modulators in CVID patients.

**Table 1:** Proposed interview to select donors for FMT [adapted from (206)]

Preliminary interview—medical history
*Drugs that can alter gut microbiota*
Recent (≤ 3 months) exposure to systemic antimicrobial drugs, immunosuppressant agents, chemotherapy
Chronic treatment (≥ 3 months) with daily use of proton pump inhibitors
*Disorders potentially associated with the disruption of gut microbiota:*
Personal history of chronic gastrointestinal disease, including functional gastrointestinal disorders; inflammatory bowel disease; celiac disease; other chronic gastroenterological diseases or recent abnormal gastrointestinal symptoms (e.g., diarrhea, hematochezia, etc.)
Personal history of cancer, including gastrointestinal cancers or polyposis syndrome, and first-degree family history of premature colon cancer
Personal history of systemic autoimmune disorders
Obesity (body mass index > 30) and/or metabolic syndrome/diabetes
Personal history of neurological/neurodegenerative disorders
Personal history of psychiatric/neurodevelopmental conditions
*Known history or risk behaviors for infectious disease*
History of HIV, hepatitis B or C viruses, syphilis, human T-lymphotropic virus I and II
Current systemic infection
Use of illegal drugs
High-risk sexual behavior
Previous tissue/organ transplant
Recent hospitalization or discharge from long-term care facilities
High-risk travel
Needle stick accident in the last six months
Body tattoo, piercing, earring, acupuncture in the last six months
Enteric pathogen infection in the last two months
Acute gastroenteritis with or without confirmatory test in the last two months
History of vaccination with a live attenuated virus in the last two months

**Table 2:** Proposed donor blood and stool testing for FMT [adapted from [256]]

Blood testing
Complete blood cell count with differential
Liver enzyme (Aminotransferases), bilirubin
Creatinine
C-reactive protein
Treponema pallidum
Nematodes (*Strongyloides stercoralis*)
Serology for Hepatitis virus (HAV, HBV, HCV, HEV) and Human immunodeficiency virus (HIV)
*Stool testing*
*Clostridioides difficile*
Common enteric pathogens, including Salmonella, Shigella, Campylobacter, shiga toxin-producing *Escherichia coli*, Yersinia and *Vibrio cholerae*
Antibiotic-resistant bacteria (ARB), including vancomycin-resistant Enterococci (VRE), methicillin-resistant *Staphylococcus aureus* (MRSA), Gram-negative ARB including extended-spectrum β-lactamase (ESBL)-producing Enterobacteriaceae, and carbapenem-resistant Enterobacteriaceae/carbapenemase-producing Enterobacteriaceae (CRE), Shiga toxin-producing E. coli (STEC)
Norovirus, rotavirus, adenovirus
*Giardia lamblia*, *Cryptosporidium* spp, Isospora and Microsporidia
Protozoa and helminths/va and parasites (including *Blastocystis hominis* and *Dientamoeba fragilis*)
*Helicobacter pylori* fecal antigen (for upper route of FMT delivery)
