# Correction to: Is there a role for microbiome‑based approach in common variable immunodeficiency?

**DOI:** 10.1007/s10238-023-01032-1

**Published:** 2023-03-14

**Authors:** Remo Poto, Gianluca Ianiro, Amato de Paulis, Giuseppe Spadaro, Gianni Marone, Antonio Gasbarrini, Gilda Varricchi

**Affiliations:** 1 Department of Translational Medical Sciences, University of Naples Federico II, 80131 Naples, Italy; 2 Center for Basic and Clinical Immunology Research (CISI), University of Naples Federico II, 80131 Naples, Italy; 3 World Allergy Organization (WAO), Center of Excellence, 80131 Naples, Italy; 4 Department of Oncology and Molecular Medicine, Istituto Superiore Di Sanità (ISS), Rome, Italy; 5 Digestive Disease Center, Fondazione Policlinico Universitario “A. Gemelli” IRCCS, Rome, Italy; 6 Department of Translational Medicine and Surgery, Catholic University of Rome, Rome, Italy; 7 Institute of Experimental Endocrinology and Oncology (IEOS), National Research Council, 80131 Naples, Italy

**Correction: Clinical and Experimental Medicine** 10.1007/s10238-023-01006-3

The original version of this article unfortunately contained a mistake. Author’s family name Ianiro was incorrectly written as Laniro.

The correct name should be

Gianluca Ianiro

